# INTERACTIVE ROLE OF SLEEP AND AROUSAL ON COGNITION AND BRAIN STRUCTURE IN CHRONIC PAIN PATIENTS

**DOI:** 10.1093/geroni/igad104.1832

**Published:** 2023-12-21

**Authors:** Ashley Curtis, Neetu Nair, Jason Craggs, Kevin McGovney, Christina McCrae

**Affiliations:** University of South Florida, Tampa, Florida, United States; University of Missouri, Columbia, Missouri, United States; University of South Florida, Tampa, Florida, United States; University of Missouri, Columbia, Missouri, United States; University of South Florida, Tampa, Florida, United States

## Abstract

Chronic widespread pain (CWP) patients experience heightened arousal, insomnia complaints, poor cognition, and diffuse brain structure alterations, but interactive relationships among symptoms remain unexplored. We assessed whether physiological arousal [assessed via heart rate variability (HRV)] moderated associations between sleep and cognition/brain structure. Adults (N=42, Mage=46.2±13.7) with CWP and insomnia (difficulty falling/staying asleep) completed 14 daily sleep diaries, 5-minute Holter monitoring [HRV root mean square of successive normal heartbeats (RMSSD), low frequency/high frequency (lf/hf) computed], cognitive tasks [Stroop (processing speed/attention/inhibition), Sternberg (working memory)], and magnetic resonance imaging. Moderation analyses examined interactive associations between sleep [total sleep time (TST), wake after sleep onset, sleep onset latency (SOL)] and HRV with cognition/gray matter volume of frontal/temporal regions. SOL and lf/hf interacted in associations with working memory and attention/processing speed. Longer SOL was associated with worse Sternberg/Stroop at lowest sympathetic predominance levels. Conversely, longer SOL was associated with better Stroop at highest lf/hf. SOL and RMSSD interacted in associations with anterior cingulate, showing longer SOL associated with lower volumes at lowest arousal levels. TST and RMSSD interacted in associations with right hippocampus, with shorter TST associated with lower volume at highest/average arousal levels. Shared dynamic physiological arousal and sleep onset hyperarousal mechanisms impact cognition and neural structure in CWP. Longer SOL may only negatively impact cognition/anterior cingulate structure up to a certain arousal threshold, with shared mechanisms potentially benefitting cognition at highest arousal levels. At higher arousal, longer TST may protect against hippocampal volume reduction. Future prospective studies are needed to inform temporal characteristics.

